# Low microsatellite instability: A distinct instability type in gastric cancer?

**DOI:** 10.1007/s00432-023-05430-6

**Published:** 2023-10-11

**Authors:** Meike Kohlruss, Shounak Chakraborty, Alexander Hapfelmeier, Moritz Jesinghaus, Julia Slotta-Huspenina, Alexander Novotny, Leila Sisic, Matthias M. Gaida, Katja Ott, Wilko Weichert, Nicole Pfarr, Gisela Keller

**Affiliations:** 1 Institute of Pathology, TUM School of Medicine, Technical University of Munich, Trogerstr. 18, 81675 Munich, Germany; 2 Institute of AI and Informatics in Medicine, School of Medicine, Technical University of Munich, Munich, Germany; 3 Institue of Pathology, University of Marburg, Marburg, Germany; 4 Department of Surgery, TUM School of Medicine, Technical University of Munich, Munich, Germany; 5 Department of General, Visceral and Transplantation Surgery, University of Heidelberg, Heidelberg, Germany; 6 Institute of Pathology, University of Heidelberg, Heidelberg, Germany; 7 Institute of Pathology, University Medical Center Mainz, JGU-Mainz, Mainz, Germany; 8 TRON-Translational Oncology at The University Medical Center of The Johannes Gutenberg University gGmbH, Mainz, Germany; 9 Department of Surgery, Klinikum Rosenheim, Rosenheim, Germany; 10 Institute of Pathology, German Cancer Consortium [DKTK], Partner Site Munich, Munich, Germany

**Keywords:** Adenocarcinoma, Gastric, Microsatellite instability, Tumor mutation burden, Neoadjuvant chemotherapy

## Abstract

**Purpose:**

We recently showed that low microsatellite instability (MSI-L) is associated with a good response to platinum/5-fluorouracil (5-FU) neoadjuvant chemotherapy (CTx) in gastric cancer. The purpose of this study was to characterize the instability pattern and to investigate an association of MSI-L tumors with mutations in genes of DNA repair pathways and with total tumor mutation burden (TMB).

**Methods:**

MSI patterns were compared between 67 MSI high (-H) and 35 MSI-L tumors. Whole-exome sequencing was performed in 34 microsatellite stable (MSS) and 20 MSI-L tumors after or without neoadjuvant CTx.

**Results:**

Of the 35 MSI-L tumors, 33 tumors had instability at a dinucleotide repeat marker. In the homologous recombination (HR) pathway, 10 of the 34 (29%) MSS and 10 of the 20 (50%) MSI-L tumors showed variants (*p* = 0.154). In the DNA damage tolerance pathway, 6 of the 34 (18%) MSS and 7 of the 20 (35%) MSI-L tumors had variants (*p* = 0.194). The HR deficiency score was similar in both tumor groups. TMB was significantly higher in MSI-L compared to MSS tumors after CTx (*p* = 0.046). In the MSS and MSI-L tumors without CTx no difference was observed (*p* = 1.00).

**Conclusion:**

MSI-L due to instability at dinucleotide repeat markers was associated with increased TMB after neoadjuvant CTx treatment, indicating sensitivity to platinum/5-FU CTx. If confirmed in further studies, this could contribute to refined chemotherapeutic options including immune-based strategies for GC patients with MSI-L tumors.

**Supplementary Information:**

The online version contains supplementary material available at 10.1007/s00432-023-05430-6.

## Introduction

Microsatellites are short, repetitive DNA sequences with a tandem repeat motif of typically one to six nucleotides (Tautz et al. 1986; Ellegren 2004). Because of their repetitive nature, these DNA sequences are easily susceptible to errors during DNA replication. The errors are usually detected and repaired by the DNA mismatch repair (MMR) system. When the function of this system is impaired in tumors, widespread length changes of the original microsatellite sequence occur, which is referred to as high microsatellite instability (MSI-H).

MSI can be determined by PCR-based fragment analysis. Standardized panels of either five markers of the so-called Bethesda panel with two mononucleotide and three dinucleotide repeat markers or five microsatellite markers comprising only mononucleotide repeats are analyzed (Boland et al. 1998, Boland et al. 2010). If at least two of the five markers are unstable, the tumor is classified as MSI-H; if only one marker is unstable, the tumor is classified as MSI-low (L).

In gastric carcinomas, MSI-H is found in relatively broad range of 8–22% of tumors and the Cancer Genome Atlas Project has identified MSI-H tumors as a distinct molecular class, associated with specific clinicopathological features (Cancer Genome Atlas Research 2014, Polom et al. 2018; Kohlruss et al. 2019). In addition, MSI-H has attracted much attention as a predictive biomarker for immune checkpoint therapy (Le et al. 2015, 2017; Chao et al. 2021).

The MSI-L phenotype has been described in several tumor types, including 5–7% of gastric carcinomas (Pawlik et al. 2004; An et al. 2012; Kohlruss et al. 2019; Imamura et al. 2021). In contrast to MSI-H, MSI-L is less well characterized, and the molecular background of this instability type is largely unknown. Moreover there is controversy as to whether MSI-L represents an independent instability type or reflects some background mutation rate and.

MSI-L and microsatellite stable (MSS) tumors are frequently considered as one group (Jass et al. 2001; Tomlinson et al. 2002; Halford et al. 2003; Pawlik et al. 2004, Cancer Genome Atlas Research 2014).

In a recent study, we evaluated MSI in 760 gastric carcinomas for prognostic significance and relevance to predict response to neoadjuvant chemotherapy, considering MSI-H and MSI-L separately. Interestingly, we found different characteristics for MSI-H and MSI-L phenotypes. MSI-H was associated with a good prognosis independent of CTx treatment, whereas MSI-L predicted a good response to platinum/5-FU based neoadjuvant CTx, but showed a negative prognostic effect for patients who received surgery alone (Kohlruss et al. 2019). The MSI-L phenotype has been associated with the expression of MSH3 in colorectal cancer (Plaschke et al. 2012). However, in a previous study, we did not find any association between the expression of MSH3 or the other classical MMR proteins and our MSI-L tumors (Herz et al. 2022).

The aim of the study presented here was to gain a more detailed insight into the molecular genetic background of the MSI-L phenotype in our tumors. Therefore, we first asked whether MSI-L is associated with a specific instability pattern. Second, using whole-exome sequencing (WES) of a subset of the tumors, we investigated whether there was a possible association with mutations in genes of specific DNA repair, DNA synthesis and DNA damage tolerance pathways and with the homologous recombination deficiency (HRD) score. Third, we asked whether MSI-L differed from MSS tumors in terms of total tumor mutational burden (TMB).

## Patients and methods

### Patients and chemotherapy

Tumors of 57 patients with gastric adenocarcinomas, including tumors of the gastro-esophageal junction (Siewert type II and III) (Siewert et al. 1998) that were treated between 2003 and 2012 at the Department of Surgery of the University of Heidelberg and between 2001 and 2013 at the Technical University of Munich were analyzed by WES in this study. Selection of the tumors was based on the MSI status with a main focus to include most of our MSI-L tumors with sufficient DNA with a good quality from formalin fixed paraffin embedded (FFPE) tumor tissues. Overall 28 patients were treated with neoadjuvant chemotherapy and the chemotherapy regimens used are listed in Supplementary Table S1.

### Ethics statement

The use of tissue samples was approved by the local Institutional Review Boards at the Technical University Munich (reference: 502/15 s) and at the University of Heidelberg (reference: 301/2001). All experiments were performed in accordance with the Declaration of Helsinki.

### Response evaluation

The response to neoadjuvant CTx was evaluated histopathologically and classified into three tumor regression grades (TRG): TRG1(a/b), TRG2, and TRG3, which corresponded to < 10%, 10–50% and > 50% residual tumor cells within the tumor bed, respectively (Becker et al. 2011). Only patients after neoadjuvant CTx with TRG2 or TRG3 were included in the study.

### Microsatellite analysis

Microsatellite analysis including DNA isolation from normal and tumor FFPE tissue were described in our previous studies (Kohlruss et al. 2019, 2021). In brief, MSI was analyzed using five markers of the Bethesda panel, encompassing two mononucleotide repeat markers, BAT25 and BAT26, and three dinucleotide repeat markers D2S123, D5S346, and D17S250, as recommended by the National Cancer Institute (Boland et al. 1998). According to a standardized definition, MSI-H was defined if at least two of the five markers showed MSI and as low (L) MSI, if only one of the five markers showed MSI. MSI-L has been confirmed by a second independent PCR and fragment analysis (Kohlruss et al. 2019). If no instabilities were observed, tumors were classified as MSS. The pattern of microsatellite instability was analyzed for insertions and deletions. In case of heterozygosity and an unstable allele between the major alleles, an insertion or deletion was defined depending on whether the length of the instability occurred closer to the major or minor allele. If this was not clearly possible, it was defined as not classifiable.

### DNA quantification and quality assessment for whole exome sequencing

DNA for WES was isolated from FFPE tissues after microdissection, deparaffinization and proteinase K digestions using the Maxwell RSC16 extraction system according to the instructions of the manufacturer (Promega, Madison, WI, USA). DNA concentration was measured fluorimetrically by the Qubit 3.0 system (Thermo Fisher Scientific, Waltham, MA, USA) and the DNA quality was determined by a qPCR assay (RNAse P assay, Thermo Fisher Scientific) as described (Endris et al. 2013).

The DNA degradation index (DDI) was calculated by dividing the quantity of amplifiable DNA calculated by the RNase P assay by the concentration of the total amount of DNA quantified by the Qubit dsDNA high-sensitivity assay. Tumors with DDI values < 0.2 were not used for sequencing.

### Whole exome sequencing

DNA was send to Genewiz (Leipzig, Germany) for WES using the Agilent SureSelect Human All Exon V6 chemistry. Sequencing was performed on an Illumina HiSeq 2 × 150 bp sequencing with a 100 × coverage. Bioinformatic analysis was performed by Genewiz encompassing the following steps: Raw BCL files generated by the sequencer were converted to fastq files for each sample using bcl2fastq v.2.19. Sequence reads were trimmed using Trimmomaticv.038. The trimmed reads were mapped to the reference genome (GRCh37, hg19) using the Illumina Dragen Bio-IT platform and BAM files were generated. Somatic variants were called using the Illumina Dragen Bio-IT platform in somatic mode. A panel of normal was used to remove technical artefacts. Variants were filtered and any variants with the following criteria were considered as false positive and were removed: marked as common variants in the db SNP build 151 and non_cancer_AC < 5 in gnomad exome database r2.1.1. The filtered VCF files were annotated with Ensemble Variant Effector Predictor (VEP) v95.

### Candidate variant selection and gene panels

We selected variants with allele frequencies > 10%, with altered read depth > 10 and based on possible functional effects, for further analysis. The latter criteria were as follows: (a) all variants with high impact by the VEP, (b) variants with moderate impact by VEP and annotated as deleterious by the SIFT algorithm and additionally classified as probable or possible damaging by the PolyPhen algorithm.

We used the R Bioconductor package “Maftools” to compare selected gene panels between the MSS and MSI-L group (Mayakonda et al. 2018). The panels were selected according to data in the literature and/or according to the Reactome database (https://reactome.org) and encompassed genes involved in a) homologous recombination repair (HR) (*n* = 149) (Lord et al. 2016), (b) Fanconi anemia pathway (*n* = 38), (c) canonical and alternative non homologous end joining (NHEJ) (*n* = 60), (d) nucleotide excision repair (NER) (*n* = 110), e) DNA synthesis (*n* = 121) and (f) DNA damage tolerance pathway including translesion synthesis, template switching and repriming (*n* = 98) (Reactome database) (Bainbridge et al. 2021). Gene lists are included in Supplementary File S1.

### Homologous recombination deficiency (HRD) score

WES reads were aligned to the human reference genome (GRCh37, hg19) using BWA MEM aligner (version 0.7.17-r1188) (Li 2013). Copy number alterations (CNA) and B-allele frequencies were estimated using the command line tool Control-FREEC (version 11.6) (Boeva et al. 2012) and HRD score along with its three constituent values, loss of heterozygosity (LOH), telomeric allelic imbalance (TAI) and number of large-scale state transitions (LST) were calculated using the R package scarHRD (Sztupinszki et al. 2018). Control-FREEC and scarHRD were run using default parameters as per their respective manuals.

### Tumor mutation burden

Tumor mutation burden (TMB) was calculated by dividing the total number of somatic variants (synonymous and nonsynonymous variants, insertions, deletions and SNV at splicing sites) per tumor exome. According to Chalmers et al., we referred to 38 Mb as the estimate of the exome size (Chalmers et al. 2017).

In addition, TMB was also calculated by dividing the total number of somatic variants (synonymous and nonsynonymous variants, insertions and deletions) per target region covered by the Agilent SureSelect Human All Exon V6, which corresponded to 60.456963 Mb.

### Statistical analysis

Chi-squared tests or Fisher's exact tests were used for hypothesis testing of the differences between relative frequencies. The Mann–Whitney *U* test was used to compare continuously scaled variables. All statistical analyzes were performed using SPSS, Version 25 (IBM Corp., Armonk, NY, USA). Exploratory 5% significance levels (two-tailed) were used for the hypothesis testing.

## Results

### Study design and patient characteristics

In our previous studies 67 MSI-H, 35 MSI-L and 613 MSS tumors of patients with gastric adenocarcinomas, had been identified and patients characteristics had been reported in detail (Kohlruss et al. 2019, 2021).

**Fig. 1:**
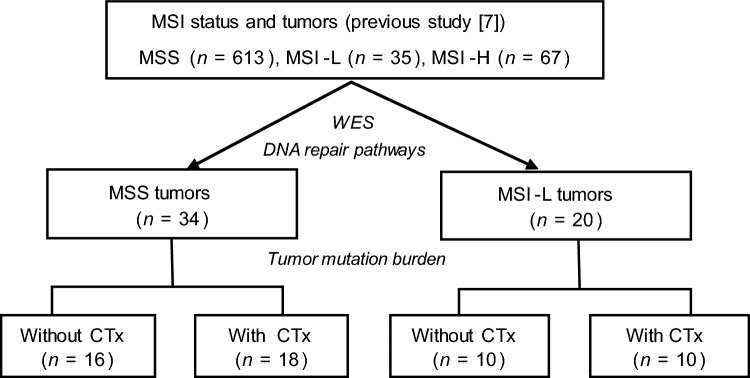
Flow diagram of tumors with MSI status and sample inclusion. The total number of tumors with their MSI status is shown. *MSI-H* high microsatellite instability, *MSI-L* low microsatellite instability, *MSS* microsatellite stable, *WES* whole exome sequencing, *CTx* chemotherapy

Tumors from 57 patients were further analyzed by WES in this study. Of the 57 patients, 34 were MSS, 20 were MSI-L and 3 were MSI-H. Criteria for selection of the 34 MSS tumors were similar proportions regarding tumor localization, histopathological type and treatment with neoadjuvant CTx in comparison to the MSI-L tumors. Three MSI-H tumors were included for control reasons. An overview of the tumors used for WES is shown in Fig. 1.

Of the 34 MSS patients, 16 (47%) were primarily resected and 18 (53%) were treated with platinum/5-FU based neoadjuvant CTx. Of the 20 MSI-L patients, 10 (50%) were primarily resected and 10 (50%) were treated with neoadjuvant CTx. Detailed patient characteristics of the MSS and MSI-L groups are shown in Table 1.

**Table 1:** Patient characteristics

		MSS tumors	MSI-L tumors
Category	Value	*n* (%)	*n* (%)
Cases	Total	**34 (100)**	**20 (100)**
Age [yr]	Median	68.4	64.8
	Range	45.2–84.6	49.3–82.2
Sex	Male	29 (85)	18 (90)
	Female	5 (15)	2 (10)
Tumor localization	Proximal	15 (44)	11 (55)
	Non proximal	19 (56)	9 (45)
Laurén classification	Intestinal	22 (65)	14 (70)
	Non-intestinal	12 (35)	6 (30)
Clinical tumor stage (cT)	cT2	9 (27)	4 (20)
	cT3/4	25 (73)	16 (80)
(y)pT^a^	(y)pT0,1,2	3 (9)	3 (15)
	(y)pT3,4	31 (91)	17 (85)
(y)pN^a^	Negative	12 (35)	4 (20)
	Positive	22 (65)	16 (80)
Metastasis status	No	29 (85)	17 (85)
	Yes	5 (15)	3 (15)
Resection category	R0	24 (71)	13 (65)
	R1	10 (29)	7 (35)
Neoadjuvant CTx	Yes	18 (53)	10 (50)
	No	16 (47)	10 (50)
Tumor regression grade	n/a	16 (47)	10 (50)
	TRG2	7 (21)	5 (25)
	TRG3	11 (32)	5 (25)

*MSS* microsatellite stable, *MSI-L* low microsatellite instability CTx, neoadjuvant chemotherapy, *n/a* not available

^a^TNM classification according to 7th Edition UICC

### Frequency and pattern of MSI

**Fig. 2:**
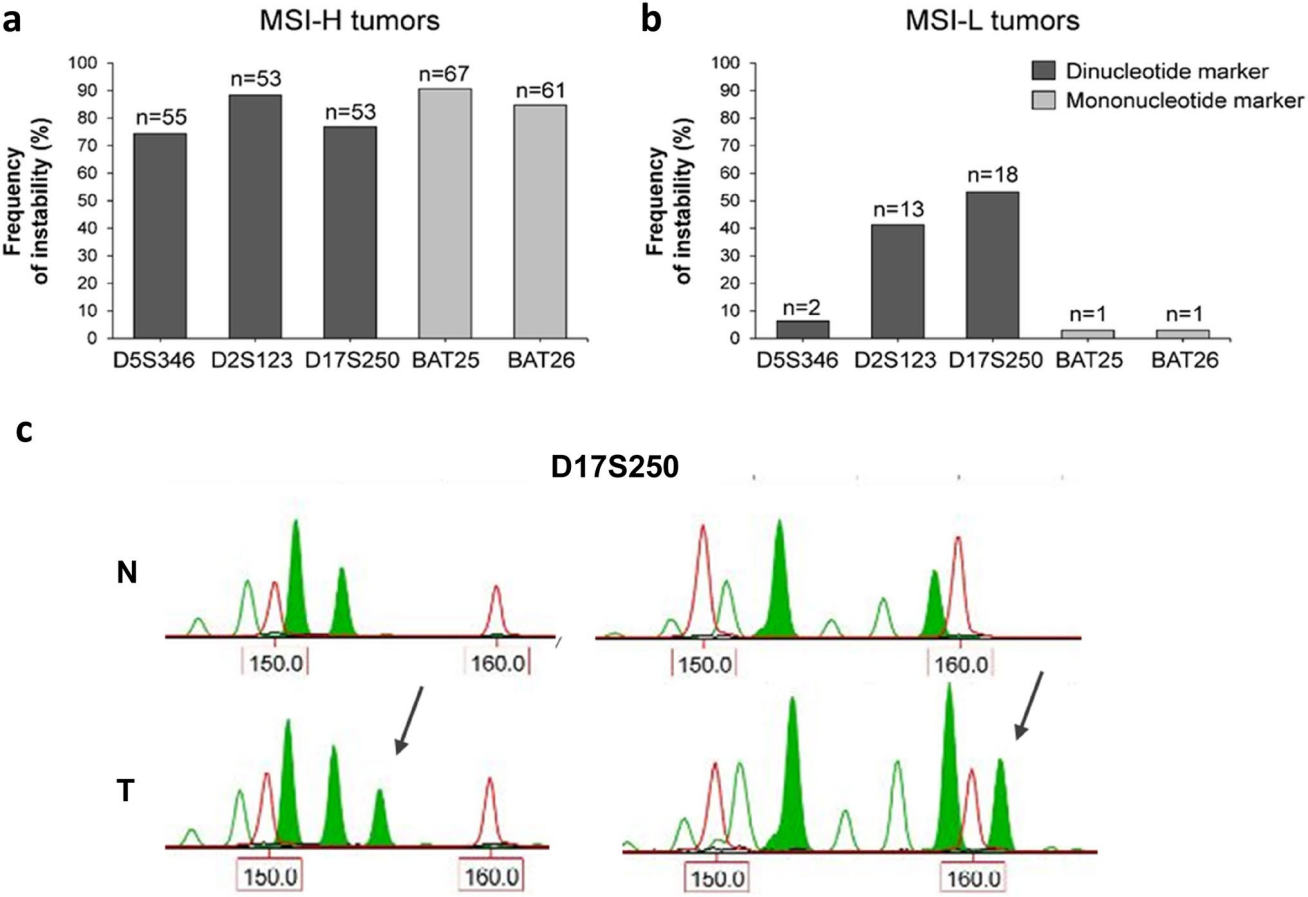
Frequency of marker instability and examples of MSI-L. Frequency of instabilities at the five markers tested in **a** MSI-H and **b** MSI-L tumors; **c** Examples of MSI-L at marker D17S250. Microsatellite alleles are shown in green. Additional alleles in the tumors are indicated by arrows. Size markers at 150 and 160 base pairs are shown in red. *N* normal, *T* tumor, *MSI-H* high microsatellite instability, *MSI-L* low microsatellite instability

As a first step, we compared the frequency of unstable microsatellite markers between MSI-H and MSI-L tumors. Among the 67 MSI-H tumors, 96% and 88% showed instability at the mononucleotide repeat markers BAT25 and BAT26, respectively. Among the dinucleotide repeats, 91% showed instability at D2S123, 75% at D5S346, and 81% at D17S250 (Fig. 2a).

Of the 35 MSI-L tumors, only two each had instability at the mononucleotide repeat markers and both were deletions. Instability at one of the dinucleotide markers was observed in 33/35 (94%) of the MSI-L tumors, with 45% of them having instability at D2S123, 9% at D5S346, and 50% at D17S250 (Fig. 2b). Of note, of the 33 tumors with instability at the dinucleotide repeats, 31 (94%) had an insertion of typically two base pairs. One tumor had a deletion and in one case the instability was unclassifiable. Examples are shown in Fig. 2c.

### Sequence variants of genes of DNA repair, DNA synthesis and DNA damage tolerance pathways and MSI status

**Fig. 3:**
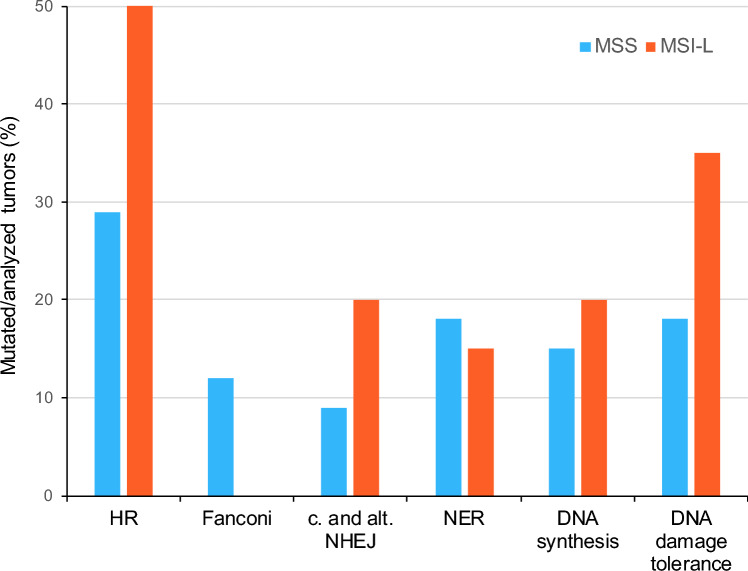
Frequencies of MSS and MSI-L tumors with sequence variants in DNA repair, DNA synthesis and DNA damage tolerance pathways. Frequencies of tumors with sequence variants in DNA repair, DNA synthesis and DNA damage tolerance pathways are shown. *HR* homologous recombination DNA repair, *Fanconi* Fanconi anemia pathway; c. and a. *NEHJ* canonical and alternative non homologous end joining, *NER* nucleotide excision repair, *MSS* microsatellite stable, *MSI-L* low microsatellite instability

We compared sequencing variants in DNA repair, DNA synthesis and DNA damage tolerance pathways between 34 MSS and 20 MSI-L tumors. Mutation frequencies ranged from 3 to 30% mutated tumors in the MSS group and from 0 to 50% mutated tumors in the MSI-L group. An overview of the mutation frequencies of the analyzed pathways is shown in Fig. 3. Detailed data are summarized in Supplementary Table S2. Overall no statistical significant differences were found, but some interesting distinction were observed.

**Fig. 4:**
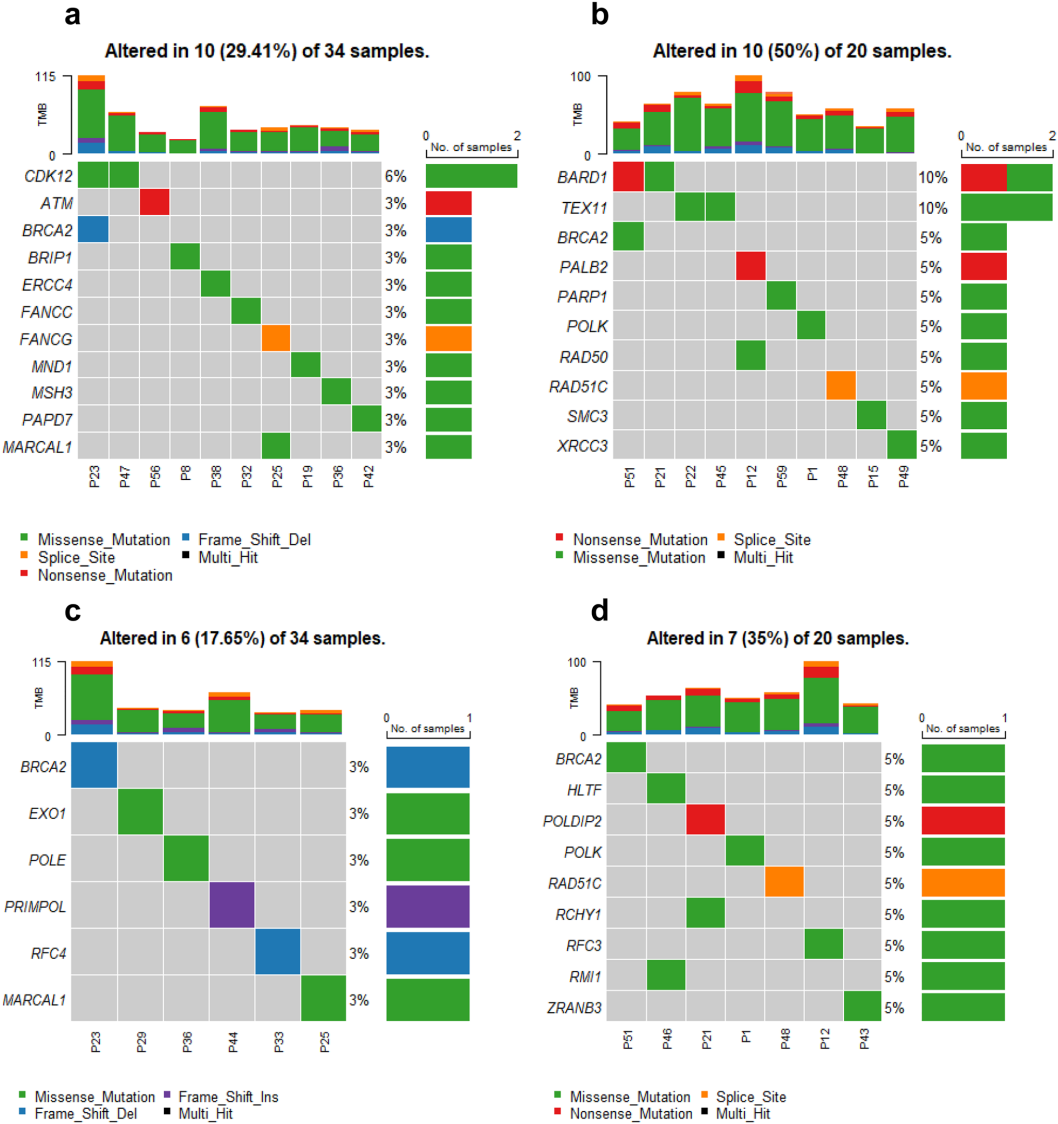
DNA sequence variants in the HR and DNA damage tolerance pathway in MSS and MSI-L tumors. Oncoplots of the sequence variants of the genes of HR in **a** MSS tumors, **b** MSI-L tumors and of genes of DNA damage tolerance pathway in **c** MSS tumors, **d** MSI-L tumors. Bars represent the mutation rate in the respective tumor group. *MSS* microsatellite stable, *MSI-L* low microsatellite instability

The frequency of mutations in the HR pathway varied between the MSS and MSI-L tumors, as 10 of the 34 (29%) MSS tumors and 10 of the 20 (50%) MSI-L tumors had variants in this gene panel (*p* = 0.154) (Fig. 4a, b).

In the Fanconi anemia pathway, 4 of the 34 (12%) MSS tumors, but no MSI-L tumor showed DNA variants (*p* = 0.285). With respect to the canonical and alternative NEHJ pathways, 3 of the 34 MSS (9%) and 4 of the 20 MSI-L tumors (20%) had variants (*p* = 0.403) (Supplementary Fig. S1a, b).

An obvious difference was observed in genes involved in the DNA damage tolerance pathway including template switching, translesion synthesis and repriming. In the MSS group, 6 of 34 (18%) and in the MSI-L group, 7 of 20 (35%) tumors had sequence variants (*p* = 0.194) (Fig. 4c, d). Slight differences between the MSS and MSI-L tumors were observed for NER and DNA synthesis pathways.

Three MSI-H tumors were included in our analysis and showed overall 19 variants in the HR gene panel. Two tumors had 6 variants in the NHEJ panel and all three showed overall 14 variants in the DNA damage tolerance panel (Supplementary Fig. S2a-c).

### Description of the identified variants

The DNA variants in the HR, NHEJ, Fanconi anemia and DNA damage tolerance pathways are listed in Supplementary File S2. In total, we identified 38 variants in these pathways, including 28 missense mutations, 3 frame shift, 4 nonsense, and 3 splice site mutations. Eighteen of the 38 variants are novel, four are listed in the COSMIC or Human Genome Mutation Database (HGMD), 11 in the SNP database, and five in two of the databases.

Considering mutations in the key molecules *BRCA2*, *RAD51C*, and *PALB2*, which are involved not only in HR but also in cNHEJ and the DNA damage tolerance pathway, three variants were identified in the MSI-L group and one *BRCA2* variant in the MSS group.

Overall, several variants were identified that are listed in the SNP database, but the minor allele frequencies of these variants are less than 10^–3^ in all cases.

### HRD score in correlation to MSI status

Values for LOH, telomeric allelic imbalance (TAI), large-scale state transition (LST), and the combined HRD score of these three parameters were similar in the MSS and MSI-L tumor groups. The median of the HRD score was 28.5 (range 4–42) in the MSS group and 27.5 (range 12–43) in the MSI-L group (*p* = 0.701). Thus, overall no significant differences were found. The detailed results are summarized in Supplementary Table S3.

### TMB, MSI status and neoadjuvant chemotherapy

**Fig. 5:**
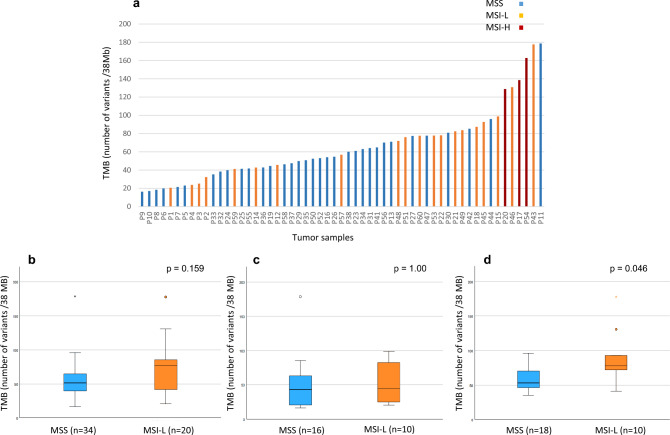
Total tumor mutation burden (TMB) in MSS and MSI-L tumors and neoadjuvant CTx. TMB per sample of MSS, MSI-L and MSI-H tumors is shown in **a**. The median TMB of MSS and MSI-L tumors is shown in **b** all tumors and in tumors stratified according to neoadjuvant CTx **c** without CTx; **d** after CTx. *p*-value: Mann–Whitney *U* test. *MSS* microsatellite stable, *MSI-L* low microsatellite instability, *CTx* chemotherapy

The TMB was found in a range of 16–179 mutations/Mb. A summary of TMB of MSS, MSI-L, and MSI-H tumors is shown in Fig. 5a.

The median TMB of MSI-L tumors was 76.87 (range 20.61–177.68) and was higher than the median of 51.66 (range 16.37–178.63) of MSS tumors (*p* = 0.159) (Fig. 5b).

Comparison of TMB between MSS and MSI-L tumors in the subgroup of patients stratified by neoadjuvant chemotherapy (yes or no) revealed a striking difference. In the group without CTx, the median TMB of the MSS tumors was 42.97 (range 16.36–178.63), which was not significantly different from the median of 44.25 (range 20.60–98.76) of the MSI-L tumors (*p* = 1.00) (Fig. 5c). However, in the CTx group, the median of 77.72 (range 41.24–177.69) in the MSI-L tumors was significantly higher than the median of 52.76 (range 35.29–95.95) in the MSS tumors (*p* = 0.046) (Fig. 5d).

Calculation of TMB including intronic variants with reference to the target region of 60.456963 Mb covered by the sequencing kit yielded equivalent results (data not shown).

## Discussion

In this study, we characterized the MSI-L phenotype in terms of the pattern of instability, the mutation spectrum of genes involved in DNA repair and DNA damage tolerance pathways, and in terms of total TMB.

The most interesting finding of our study was the association of MSI-L with a higher TMB specifically in the tumors after treatment with neoadjuvant CTx. This suggests that the MSI-L phenotype reflects higher susceptibility to the chemotherapeutic agents with which the patients were treated, namely cis/oxaliplatin and 5-FU. In a previous study, we had examined tumor biopsies from gastric cancer patients prior to neoadjuvant treatment and found a significant association of the MSI-L phenotype with good response in terms of tumor shrinkage or tumor regression (Kohlruss et al. 2019). Thus, taken together, the results support the finding that MSI-L indicates sensitivity to platinum/5-FU based CTx.

Regarding mutations in the pathways studied, more sequence variants were found in HR and DNA tolerance pathways in MSI-L tumors compared to MSS tumors. HR is the main pathway for repairing DNA double-strand breaks that can occur after chemotherapeutic treatment with platinum compounds (Rottenberg et al. 2021). In MSI-L tumors, we identified DNA variants in *BRCA2, RAD51C*, and *PALB2*, among others, which are three key molecules in this repair pathway. Mutations in these genes lead to HRD and increased sensitivity to platinum-based CTx in several tumor types (Telli et al. 2018; Nguyen et al. 2020; Wagener-Ryczek et al. 2021; Ter Brugge et al. 2023). In addition to mutations in these specific genes, HRD may also be due to alterations in other genes involved in this complex repair mechanism, resulting in specific genomic scars that include loss of heterozygosity (LOH), telomeric allelic imbalance (TAI), and large-scale state transitions (LST). Quantitative scores for LOH, TAI, and LST have been developed, as well as a combined HRD score for the three parameters that are highly correlated with HRD in breast and ovarian cancer and sensitivity to platinum agents (Timms et al. 2014; Wagener-Ryczek et al. 2021; Rempel et al. 2022). But to this date it is unclear whether the specific HRD score cut-off, which was determined only on ovarian and breast cancer, can be transferred to other tumor entities or not. Interestingly, however, when calculating the combined HRD and LOH, TAI, and LST scores in our study, we did not detect any apparent differences between the MSS and MSI-L tumors, questioning a major role of HRD in the occurrence of MSI-L in our tumors.

As mentioned earlier, we also observed some preponderance of mutations in genes involved in DNA damage tolerance, which includes translesion synthesis, template switching of fork reversal and repriming. DNA-damaging agents such as cisplatin can also create obstacles to DNA replication leading to impaired replication fork progression, commonly referred to as replication stress and the DNA damage tolerance pathway is important to ensure adequate DNA replication fork progression (Quinet et al. 2021, Cybulla et al. 2023). This complex pathway requires the *RAD51C* recombinase and specific fork remodelers such as *HLTF* and *ZRANB3*, all of which have sequencing variants in our MSI-L tumors. In this context, it is worth noting that *RAD51C* and *BRCA2* also play important roles in protecting stalled replication forks independently of their classical function in HR (Stok et al. 2021). Of note, loss of replication fork protection in cells amenable to HR is associated with increased cisplatin sensitivity and genome instability (Mukherjee et al. 2019; Stok et al. 2021).

It is also important to note that repetitive DNA sequences such as microsatellites can form secondary structures that interfere with proper DNA replication leading to a fork stalling and collapse (Polleys et al. 2017). It could be possible that the overwhelming number of insertions we observed in the unstable microsatellite markers reflects the malfunction of a specific step that is essential for DNA replication fork progression.

Overall, one could speculate that the MSI-L phenotype is related to an impaired DNA damage tolerance pathway, at least in a subset of MSI-L tumors. We are aware that this hypothesis is highly speculative, especially given the small number of tumors analyzed. We consider this a major limitation of our study, and caution should be exercised in the final interpretation of the data. The analysis of a larger number of cases and the determination of possible clinical confounding factors is important. Therefore, we emphasize the exploratory nature of our study, which can be considered hypothesis-generating and requires further analysis in larger cohorts. In particular, comparison of mutation frequencies and patterns in corresponding biopsies and resected specimens after CTx in the same patients is crucial and could elucidate the role of DNA damage tolerance pathway in more detail. A preferential insertion of repeat units has been described for a yeast mutant of the rth1 gene, which corresponds to the flap endonuclease FEN1 in humans (Johnson et al. 1995; Polleys et al. 2017). The FEN1 gene was present in several gene panels in our study, but did not exhibit DNA variants in the tumors.

Another point we would like to address is that among the sequencing variants identified in our study, there were several very rare variants listed in the SNP database. The aim of our study was to characterize somatic variants in the tumors and it was not designed to distinguish possible very rare germline from somatic variants by sequencing in parallel the normal DNA of each patient. Thus, further appropriately designed studies have to be performed to answer this question.

The existence of a specific MSI-L phenotype has been questioned in numerous studies, and MSI-L and MSS tumors are often considered as one group (Jass et al. 2001; Tomlinson et al. 2002; Halford et al. 2003; Pawlik et al. 2004, Cancer Genome Atlas Research 2014). In our study, we used the Bethesda panel to determine MSI status, and the classification of MSI-L refers almost exclusively to instability at dinucleotide microsatellite repeats. However, MSI is also commonly analyzed using five mononucleotide repeat markers. Thus, it is clear that the determination of MSI-L by the different microsatellite marker panels can describe completely different tumors and complicates the comparison of the data in the literature.

In conclusion, our data show an association of MSI-L due to instability at dinucleotide repeat markers with increased mutational burden specifically after neoadjuvant chemotherapy, which supports the view that MSI-L indicates sensitivity to platinum/5-FU based CTx. If confirmed in further studies, this could contribute to refined chemotherapy strategies including immune-based strategies specifically for gastric cancer patients with MSI-L in their tumors.

## Supplementary Information

Below is the link to the electronic supplementary material.

Supplementary S1 Gene panels selected for the anaylsis (XLSX 25 KB)

Supplementary S2 DNA sequence variants. (XLSX 15 KB)

Supplementary Information with Supplementary Fig. S1, Fig. S2 and Supplementary Table S1 and S2 (DOCX 200 KB)

## Data Availability

Study data, which are not included in the published article and its Supplementary information files can be made available from the corresponding author on reasonable request.
