# Supplementary material for: Low microsatellite instability: A distinct instability type in gastric cancer?
Source: J Cancer Res Clin Oncol. 2023 Oct 11;149(20):17727–37. doi: 10.1007/s00432-023-05430-6 (PMC10725348; doi:10.1007/s00432-023-05430-6)
Supplement: Supplementary file 3 — Supplementary Information with Supplementary Fig. S1, Fig. S2 and Supplementary Table S1 and S2 (DOCX 200 KB) [file 432_2023_5430_MOESM3_ESM.docx]

**Supplementary information**

**Low microsatellite instability – a distinct instability type in gastric cancer?**

Meike Kohlruss, Shounak Chakraborty, Alexander Hapfelmeier, Moritz Jesinghaus, Julia Slotta-Huspenina, Alexander Novotny, Leila Sisic, Matthias M. Gaida, Katja Ott, Wilko Weichert, Nicole Pfarr, and Gisela Keller

**Supplementary Figures**


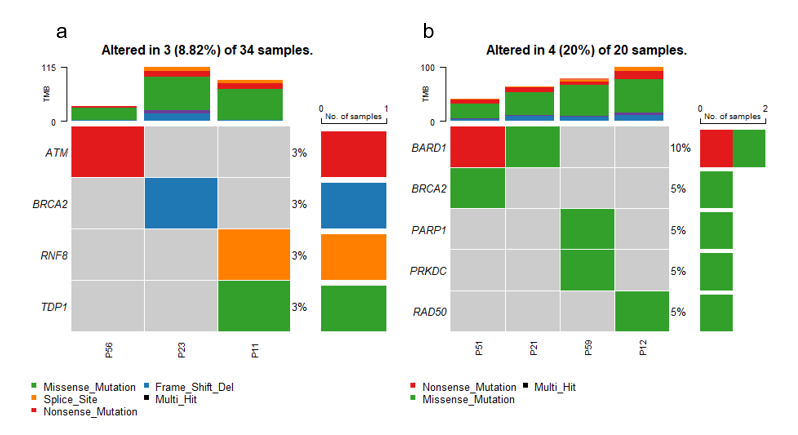


**Supplementary Fig. S1 DNA sequence variants in genes of canonical and alternative NHEJ in MSS and MSI-L tumors**

Oncoplots of the sequence variants of the genes involved in DNA repair by the canonical and alternative NHEJ pathway are shown a) MSS tumors, b) MSI-L tumors. Bars represent the mutation rate in the respective tumor group. MSS, microsatellite stable; MSI-L, low microsatellite instability; NHEJ, non homologous end joining.


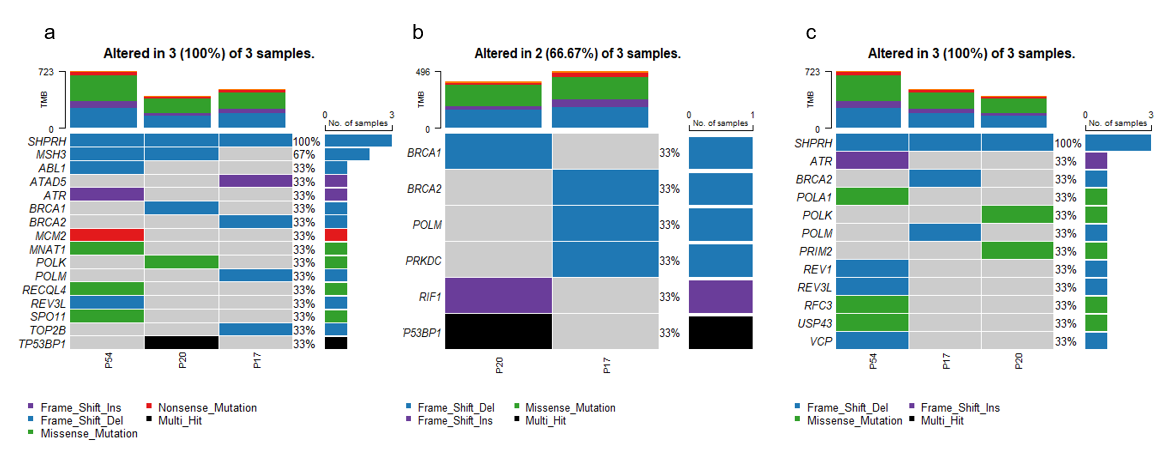


**Supplementary Fig. S2 DNA sequence variants in MSI-H tumors**

Oncoplots of the DNA sequence variants in the three MSI-H tumors are shown for genes involved in a) HR, b) canonical and alternative NHEJ, c) DNA damage tolerance pathway.

MSI-H, high microsatellite instability; HR, homolgous recombination repair; NHEJ, non homologous end joining.

**Supplementary Tables**

**Supplementary Table S1 Chemotherapy regimens**

| Neoadjuvant chemotherapy | n % | |
| --- | --- | --- |
| Total | 28 | 100 |
| Cis + 5-FU or Cap | 12 | 43 |
| Ox + 5-FU or Cap | 5 | 18 |
| Cis or Ox + 5FU or Cap + Epi | 9 | 32 |
| Cis or Ox + Ab | 1 | 4 |
| Cis+5-FU + Doc or Pac | 1 | 4 |

Cis, cisplatin; Ox, oxaliplatin; 5-FU, 5-fluorouracil; Cap, capecitabine;

Epi, epirubicin; Ab, antibody; Doc, doxetacel; Pac, paclitaxel

**Supplementary Table S2 Mutation frequencies of MSS and MSI-L tumors**

| Pathway | Number of  analysed genes | Number of mutated tumors /  analysed tumors | | p value* |
| --- | --- | --- | --- | --- |
|  |  | MSS  *n* = 34 (%) | MSI-L  *n* = 20 (%) |  |
| HR | 149 | 10 (29) | 10 (50) | 0.154 |
| Canonical and alternative NHEJ | 60 | 3 (9) | 4 (20) | 0.403 |
| NER | 110 | 6 (18) | 3 (15) | 1.000 |
| Fanconi Anemia | 38 | 4 (12) | 0 (0) | 0.285 |
| DNA synthesis | 121 | 5 (15) | 3 (15) | 1.000 |
| DNA damage tolerance | 98 | 6 (18) | 7 (35) | 0.194 |

* p value of Fisher exact test; HR, homologous recombination repair; NHEJ, non homologous end joining; NER, nucleotide excision repair;

**Supplementary Tables S3 LOH, TAI, LST and HRD score in MSS and MSI-L tumors**

| Category | Median (range) | | p value* |
| --- | --- | --- | --- |
|  | MSS *n* = 32 | MSI-L *n* = 18 |  |
| LOH** | 0.5 (0-8) | 1.0 (0-6) | 0.991 |
| TAI*** | 22.5 (4-35) | 23.0 (11-39) | 0.606 |
| LST**** | 2.0 (0-8) | 1.0 (0-12) | 0.510 |
| HRD score | 28.5 (4-42) | 27.5 (12-43) | 0.701 |

* Mann Whitney test: ** LOH: loss of heterozygosity; ***TAI: telomeric allelic imbalance

****LST: large-scale state transition; HRD score; homologous recombination deficiency score.

**Supplementary Files**

**Supplementary File S1. Gene panels selected for the analyses**

**Supplementary File S2. DNA sequence variants in the HR, NHEJ, Fanconi anemia and DNA damage tolerance pathways**
